# Breast Cancer Prevention: The Key Role of Population Screening, Breast Self-Examination (BSE) and Technological Tools. Survey of Italian Women

**DOI:** 10.1007/s13187-023-02327-3

**Published:** 2023-07-04

**Authors:** Luana Conte, Giorgio De Nunzio, Roberto Lupo, Matilde Mieli, Alessia Lezzi, Elsa Vitale, Maria Chiara Carriero, Antonino Calabrò, Maicol Carvello, Ivan Rubbi, Massimo Federico

**Affiliations:** 1 Laboratory of Biomedical Physics and Environment, Department of Mathematics and Physics “E. De Giorgi”, University of Salento, Lecce, Italy; 2 Laboratory of Interdisciplinary Research Applied to Medicine, University of Salento, Local Health Authority, Lecce, Italy; 3 “San Giuseppe da Copertino” Hospital, Local Health Authority, Lecce, Italy; 4 C.R.A.P. Comunità Riabilitativa Assistenziale Psichiatrica, Taurisano, Italy; 5 ANT Italia ONLUS Foundation (National Cancer Association), Lecce, Italy; 6 Department of Mental Health, Local Health Authority, Bari, Italy; 7 Psychologist and Psychotherapist Freelance, Milan, Italy; 8 “Nuovo Ospedale Degli Infermi” Hospital, Local Health Authority, Biella, Italy; 9 Brisighella Community Hospital, Local Health Authority, Romagna, Italy; 10 School of Nursing, University of Bologna, Faenza, Italy; 11 Breast Unit City of Lecce Hospital, Gruppo Villa Maria (GVM) Care & Research, Lecce, Italy

**Keywords:** Breast Cancer, Prevention, Breast Self Examination, Screening Programs, Apps for Screening

## Abstract

Breast cancer is the most common tumor among women worldwide and still remains the leading cause of death in women in Italy. Although survival from this pathology has increased, this disease and its treatment can have lasting or delayed effects that can greatly affect a woman's quality of life. Primary and secondary prevention are currently the best strategies to combat this cancer: improved lifestyle, early adherence to screening, Breast Self-Examination (BSE), and even now the use of technology, have become among the most important tools to ensure increasingly early diagnosis of this disease, which is a major cause of suffering and premature mortality in women. Indeed, early diagnosis of the disease can lead to a good prognosis and a high survival rate. This study investigates the attitude of Italian women to perform clinical checkups aimed at cancer prevention, particularly adherence to free screening programs offered by the National Health Service (NHS) for women in the 50–69 age group. The knowledge, use and emotional approach toward BSE as a screening tool and the use of dedicated apps for this purpose are also investigated. Low adherence to screening programs, lack of BSE practice, and nonuse of dedicated apps are just some of the results observed in this study. Therefore, it becomes essential to spread the culture of prevention, cancer awareness and the importance of screening throughout life.

## Introduction

Breast cancer is currently the most common malignant tumor in women worldwide [2, 4]. With 55,000 new diagnoses in 2020 and 12,500 deaths expected in 2021, this cancer accounts for 30% of all cancers diagnosed in Italy [1].

The risk of developing breast cancer correlates with increasing age [1] and is associated with genetic, endocrine, dietary, environmental factors, lifestyle habits, and previous breast disease, although more than half of the cases, however, cannot be attributed to any known risk factor [6, 9, 13, 14, 18, 22].

Unfortunately, despite breast cancer awareness, public attention, and advances in breast imaging for early detection, it still remains the leading cause of cancer death in women in Italy [1]. Early diagnosis, therefore, is the most important tool to intervene in time and improve the prognosis of patients. Mammography screening is a periodic secondary prevention activity aimed at women in order to make an early stage diagnosis of breast cancer and, therefore, offer less aggressive and more effective treatments, with the aim of reducing mortality from breast cancer [1]. Mammography is still considered the most effective screening test, and the organized, population-based modality is preferred over spontaneous initiative [1]. In Italy, in accordance with the guidelines concerning prevention, diagnosis, and care in oncology and in line with the standards adopted by other European countries, free mammography screening is to be offered every two years to all women aged 50–69 years [1]. In addition, in some regions, the effectiveness of screening is also being tested in a wider age range of 45 to 74 years old [1]. However, this offering is not always accepted, and geographic differences have been found to be particularly significant in terms of screening program implementation, incidence, and survival of breast cancer [16, 17]. In addition, in recent years the limitations imposed by the pandemic have not made it easier for women to move between regions, but distancing, fear of physical proximity, and unease due to protective mask wearing during an outpatient visit have also played their part. In this rather multifaceted scenario, the rise and use of smartphone Apps and e-learning courses were found to be useful and strongly recommended to educate and improve women's health beliefs and performance in breast self-examination [3, 19].

Another prevention methodology, debated but still widely recommended, is the practice of self-palpation or Breast Self-Examination (BSE), a self-examination of the breasts that any woman, starting generally in her 20’s, may choose to perform on herself monthly or occasionally to assess visible, palpable changes and report them later to her doctor. Even women who underwent breast surgery, are pregnant or breastfeeding can perform it without risk [20]. Self-palpation is the first breast cancer "prevention" tool. This simple self-assessment test allows to learn to recognize the structure and general appearance of the breast, so that early and unusual changes from the basic physiognomy of the breast can be caught, but only regular use allows to promptly detect changes and thus to best express the usefulness of BSE.

In an increasingly difficult scenario, timely adherence to screening, self-examination to detect every slightest change, and the use of technology have become among the most important tools for increasingly early diagnosis of breast cancer, which is a major public health problem, a major cause of human suffering and premature mortality among women [5]. In light of these premises, this study investigates the attitude of Italian women to perform clinical screening for the prevention of breast cancer, particularly the participation of women in the 50–69 age group in screening programs offered free of charge by the National Health Service (NHS). The knowledge, use and emotional approach of women toward breast self-examination as an investigative tool and the use of apps dedicated to it is also investigated.

## Methods

### Design

From March 2021 to January 2022 a survey was administered among Italian female population. 2375 subjects agreed to participate in the study. The survey was conducted by means of an anonymous questionnaire distributed on a voluntary basis. All women belonging to the Italian population, aged between 20 and 69 years and who agreed to participate in the study by signing the informed consent were included. Those who did not meet the inclusion criteria were excluded. Women with non-Italian citizenship were also not included. All sections of the questionnaire were computerized through the use of a preset form from the Google Drive platform, and the study was conducted through electronic dissemination. Facebook groups of various types and Instagram pages used for publishing computerized questionnaires were contacted. The sampling used was (virtual) snowball sampling until data saturation.

### Survey Instrument

The questionnaire was constructed 'ad hoc'. It consists of 32 items divided into 4 sections: the first section (5 items) contains socio-demographic data (age, geographical area in which she lives, marital status, level of education, employment status), and is followed by a second section (8 items) in which clinical controls aimed at breast cancer prevention are investigated, with particular attention to the adherence of women invited to participate in free screening offered by the NHS. The third section (16 items) assesses women's approach to and consideration of self-palpation, and finally a fourth section (4 items) explores the knowledge and use of dedicated self-palpation apps.

### Statistical Analysis

The answers to the questionnaire items of all respondents were reported using descriptive statistics. To identify items associated with differences in behavior toward breast cancer, the subjects were divided into 2 groups: The first group represents the general population (Group A, *n* = 2235) while the second group represents women who have or had breast cancer in the past (Group 2, *n* = 140).

For each question, where appropriate, respondents were also divided by age, educational level, geographic area and marital status. Continuous variables were summarized using mean and standard deviation (SD) and categorical variables using frequencies and percentages. The Mann–Whitney U-test was used for assessing difference between Groups. Possible associations between Groups (outcome) and socio-demographics data (explanatory variables) were tested by linear logistic regression. A p value < 0.05 was considered statistically significant. The statistical analyses were conducted for all qualitative and quantitative variables using Matlab software.

## Results

### Sample Demographics and Baseline Characteristics

A total of 2375 women agreed to participate in the study. Baseline characteristics were collected and reported in section 1 of the questionnaire (Table 1). Among the respondents, 338 (14%) were over 50 years old. Most of the participants (53%, *n* = 1256) were from southern Italy and the Islands. The prevalence of the sample under study was unmarried women (54%, *n* = 1280) and students (31%, *n* = 746); this is in line with the prevalent young age present in the sample under study (45% of the women were under 30 years old, *n* = 1076).

**Table 1:** Baseline characteristics and the questionnaire items of all respondents divided by the four sections. Section 2 of the Questionnaire is related to screening adhesion for all women aged 50–69 only. Possible association between Groups (outcome) and socio-demographics data (explanatory variables) such as Age, Level of education and Geographical area) were also evaluated for Sect. 2. A *p* value < 0.05 was considered statistically significant (*p < 0.05; **p < 0.01; ***p < 0.001)

Section 1: Baseline characteristics	*N*		%
Age (y)			
20–29	1076		45
30–39	544		23
40–49	417		18
50–59	249		10
60–69	89		4
Geographic area			
North	607		26
Center	512		22
South/Islands	1256		53
Marital status			
Married	944		40
Divorced	81		3
Maiden	1280		54
Separate	46		2
Widow	24		1
Education level			
Degree	954		40
High school graduation	1197		50
Junior high school diploma	202		9
Elementary license	17		1
None	5		0
Employment status			
Craftsman	254		11
Public Administration	624		26
Services/Tertiary	356		15
Student	746		31
Retired	50		2
Unemployed	345		15
Questionnaire items	*N*	%	
Section 2: Participation in screening programs (Women aged 50–69 only)			
Q1. Have you ever undergone clinical screening for early detection of breast cancer?			
Never	20	6	Age: < 0.001***
Rarely	45	13	Education level: < 0.001***
Occasionally	17	5	Geographical area: < 0.001***
Often	78	23	
Always	178	53	
Q2. If yes, please indicate the frequency			
I have never had a screening exam	17	5	Age: < 0.001***
Every two years	140	41	Education level: < 0.001***
Once a year	164	49	Geographical area: < 0.001***
Every six months	15	4	
Once a month	2	1	
Q3. Have you ever taken advantage of the region's free checkups?			
No	97	29	Age: < 0.001***
Yes	235	70	Education level: < 0.001***
I don't know what this is about	6	2	Geographical area: < 0.001***
Q4. Have you ever undergone a biopsy?			
No	151	45	Age: < 0.001***
Yes	56	17	Education level: < 0.001***
missing	131	39	Geographical area: < 0.001***
Q5. Have you ever undergone mammography?			
No	6	2	Age: < 0.001***
Yes	323	95	Education level: < 0.001***
missing	9	3	Geographical area: < 0.001***
Q6. Have you ever undergone ultrasonography?			
No	51	15	Age: < 0.001***
Yes	235	70	Education level: < 0.001***
missing	52	15	Geographical area: < 0.001***
Q7. Have you ever undergone magnetic resonance imaging (MRI)?			
No	169	50	Age: < 0.001***
Yes	25	7	Education level: < 0.001***
missing	144	43	Geographical area: < 0.001***
Section 3: Approach toward self-examination (BSE)			
Q8. How often do you perform breast self-examination?			
Never	445	19	Age: < 0.001***
Rarely	567	24	Education level: < 0.001***
Occasionally	717	30	Geographical area: < 0.001***
Often	560	24	
Always	86	4	
Q9. If no, state the reason			
I perform it	989	42	Age: < 0.001***
I perform other medical examinations	36	2	Education level: < 0.001***
I don't remember to run it	425	18	Geographical area: < 0.001***
Fear of ominous prognosis	117	5	
I don't know how to execute it correctly	807	34	
missing	1	0	
Q10. When I perform self-examination I am taking care of myself			
Strongly agree	1422	60	Age: < 0.001***
agree	849	36	Education level: < 0.001***
uncertain	99	4	Geographical area:0.09
disagree	3	0	
strongly disagree	2	0	
Q11. Self-palpation is embarrassing			
Strongly agree	32	2	Age: < 0.001***
agree	93	4	Education level: < 0.001***
uncertain	143	6	Geographical area: < 0.05*
disagree	842	35	
strongly disagree	1264	53	
Q12. Self-palpation takes too long			
Strongly agree	12	1	Age: < 0.001***
agree	53	2	Education level: < 0.001***
uncertain	302	13	Geographical area: < 0.001***
disagree	1136	48	
strongly disagree	872	37	
Q13. It is difficult to remember to do breast checks			
Strongly agree	59	2	Age: < 0.001***
agree	357	15	Education level: < 0.001***
uncertain	382	16	Geographical area: < 0.001***
disagree	932	39	
strongly disagree	645	27	
Q14. I have no privacy to perform the breast check			
Strongly agree	10	0	Age: < 0.001***
agree	85	4	Education level: < 0.001***
uncertain	152	6	Geographical area: < 0.05*
disagree	988	42	
strongly disagree	1160	48	
Q15. My breasts are too big by self-examination			
Strongly agree	23	1	Age: < 0.001***
agree	80	3	Education level: < 0.001***
uncertain	304	13	Geographical area: < 0.001***
disagree	1006	42	
strongly disagree	962	41	
Q16. I have more important problems than self-palpation			
Strongly agree	8	0	Age: < 0.001***
agree	34	1	Education level: < 0.001***
uncertain	113	5	Geographical area: 0.06
disagree	944	40	
strongly disagree	1276	54	
Q17. Can I perform self-examination correctly			
Strongly agree	299	13	Age: < 0.001***
agree	965	41	Education level: < 0.001***
uncertain	876	37	Geographical area: < 0.001***
disagree	181	8	
strongly disagree	54	2	
Q18. Would like more information about self-examination			
No	333	14	Age: < 0.001***
Yes	2042	86	Education level: < 0.001*** Geographical area: < 0.001***
Q19. Do you find the general practitioner useful for info on self- examination			
No	628	26	Age: < 0.001***
Yes	1747	74	Education level: < 0.001*** Geographical area: < 0.001***
Q20. Do you find the oncologist useful for info on self-examination			
No	459	19	Age: < 0.001***
Yes	1916	81	Education level: < 0.001*** Geographical area: < 0.05*
Q21. Do you find the nurse useful for info on self-examination			
No	1212	51	Age: < 0.001***
Yes	1162	49	Education level: < 0.001*** Geographical area: < 0.001***
Q22. Do you find the psychologist useful for info on self-examination			
No	2083	88	Age: < 0.001***
Yes	292	12	Education level: < 0.001*** Geographical area: < 0.05*
Q23. Do you find the breast specialist useful for info on self-examination			
No	67	3	Age: < 0.001***
Yes	2308	97	Education level: < 0.001*** Geographical area: < 0.05*
SECTION 4: KNOWLEDGE AND USE OF APPS DEDICATED TO BSE			
Q24. Do you know or use dedicated applications for self-palpation?			
No	2325	98	Age: < 0.001***
Yes	50	2	Education level: < 0.001*** Geographical area: < 0.05*
Q25. Use BreastTest			
I do not use any application	273	11	Age: < 0.001***
Never	775	33	Education level: < 0.01**
Rarely	31	1	Geographical area: 0.27
Occasionally	14	1	
Often	3	0	
Always	3	0	
missing	1276	54	
Q26. Do you use Igyno?			
I do not use any application	270	11	Age: < 0.001***
Never	765	32	Education level: < 0.01**
Rarely	36	2	Geographical area: 0.15
Occasionally	15	1	
Often	4	0	
Always	3	0	
missing	1282	54	
Q27. Do you use Breast Cancer Indicators?			
I do not use any application	269	11	Age: < 0.001***
Never	774	33	Education level: < 0.001***
Rarely	27	1	Geographical area: < 0.05*
Occasionally	13	1	
Often	3	0	
Always	1	0	
missing	1288	54	

### Questionnaire Items

The questionnaire items were evaluated for all respondents and data were collected (Table 1, in sections 2–4).

**Fig. 1:**
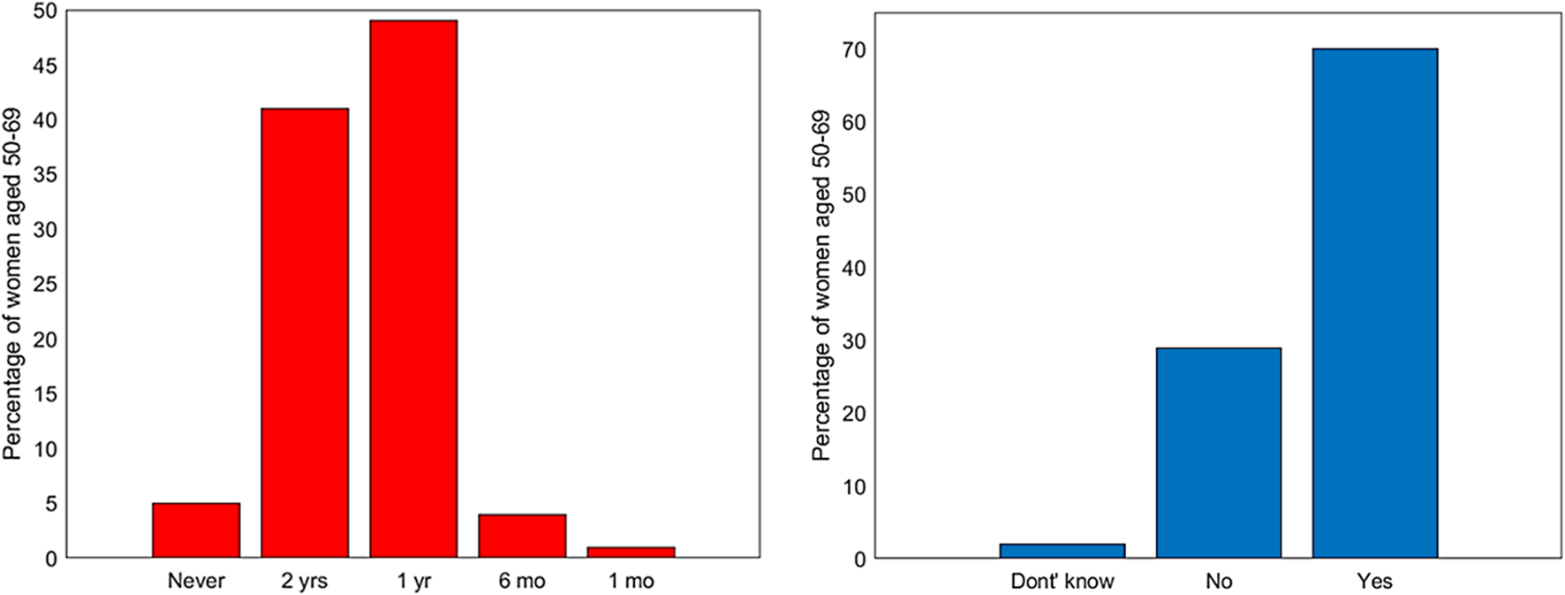
Left: frequencies of clinical controls for breast cancer for women aged 50–69 years; right: adherence to free screening program for women aged 50–69 years old

**Fig. 2:**
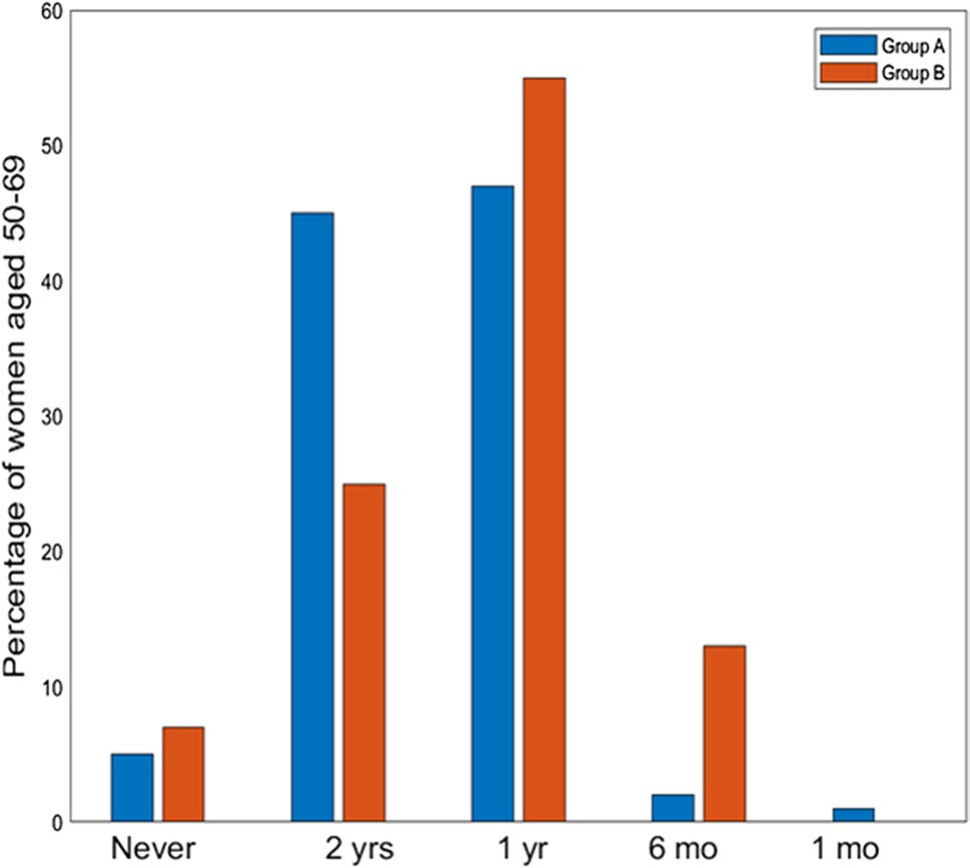
Frequencies of clinical controls for breast cancer for women aged 50–69 years divided into Group A (women of the general population) in Blue (left bars), and Group B (women already been diagnosed with breast cancer) in Orange (right bars). (yrs = years, yr = year, mo = months)

Section 2 investigates women's participation and frequency of clinical checkups performed for breast cancer prevention, with a special focus on adherence to free screenings offered by the NHS targeting women in the 50–69 age group. Surprisingly, only 53% of women over 50 say they always (*n* = 178) or often (23%, *n* = 78) participate to screenings. Specifically, 49% once a year, 41% every two years, 4% every 6 months, and 1% once a month (Fig. 1a). In addition, although almost all the women (98%) appear to be aware of the existence of free screenings offered by the NHS, 29% say they do not adhere to them (Fig. 2).

Of the examinations performed, 95% involved mammograms (*n* = 323) and 70% involved ultrasounds (*n* = 235), and this is consistent with the screening protocol that provides mammography examination for women over 50 and recommendation for ultrasound instead for younger women.

In order to test whether these data are also confirmed among women with prior breast cancer compared to other women in the general population, respondents aged 50–69 were classified into two groups: Group A including women who had not been diagnosed with breast cancer (82%, *n* = 278) and Group B including women who had already been diagnosed with breast cancer (18%, *n* = 60). As expected, looking at the participation in clinical checkups between these two groups aged 50–69 years (Table 2), significantly fewer (*p* < 0.05) women in Group A (51%) say they always perform preventive checkups compared to Group B (62%). Frequency in checkups is also higher in women with prior cancer, who report checking every six months/year in contrast to other women, who tend to have checkups mainly every two years (Fig. 2). This trend appears to be correlated with educational level (*p* = 0.001) and geographical area of origin (*p* = 0.001) (Table 1), with the South and Islands characterized by the lowest adherence.

Verifying the same data in women under 50 (data not shown), it is interesting to highlight that there are many women in the 41–50 age group who undergo mammography despite not yet being called by NHS.

Women with prior cancer, especially, check themselves annually compared with their peers, and all of them, 100% of the time, report having undergone at least one ultrasound.

**Fig. 3:**
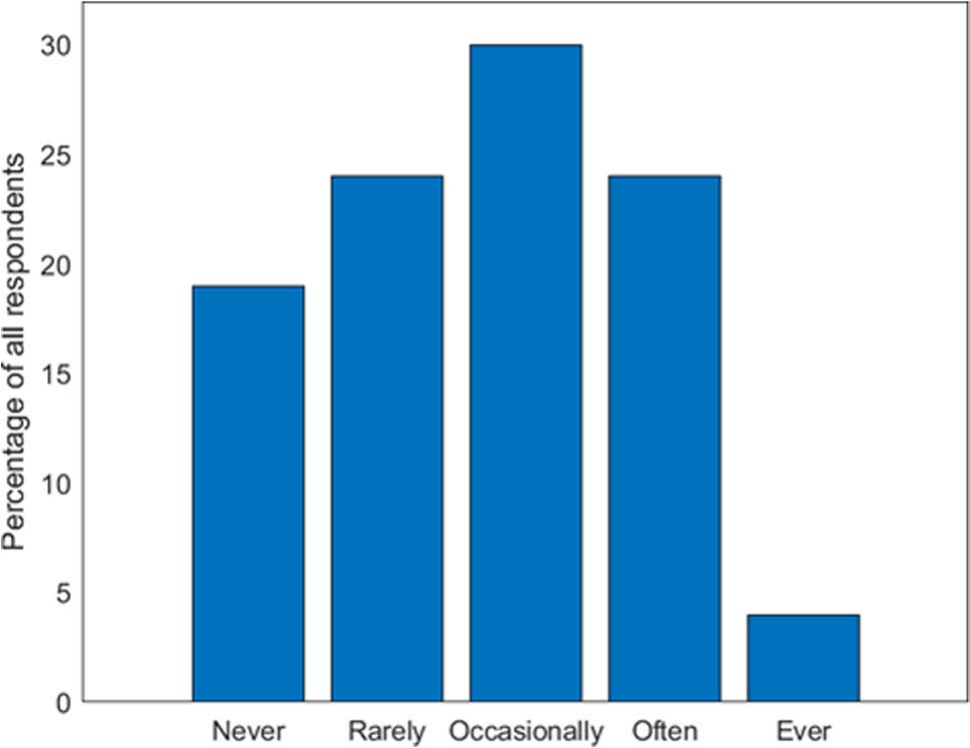
Frequencies of BSE for all respondents

Section 3 of Table 1 assesses the approach of all respondents—regardless of age—to BSE. Although 96% of respondents (*n* = 2271) agree or strongly agree that self-examination is important for women's care, only 4% say they always perform it (*n* = 86) (Fig. 3). Nearly half of women also admit that they do not know how to perform self-palpation correctly or are uncertain about it (47%, *n* = 1111). This type of insecurity or uncertainty seems to diminish with advancing age and is more characteristic of women under 50 (86%, *n* = 948) and surprisingly typical of college graduates/graduates (90%, *n* = 995).

Among those who say they do not perform it, 18% (*n* = 425) say they forget, 5% (*n* = 117) are afraid of an inauspicious prognosis, and 2% (*n* = 36) perform other medical tests.

There are also many respondents who would like more information about BSE (86% *n* = 2042), and the referral figures considered useful in order of preference are the breast specialist (97%, *n* = 2308), oncologist (81%, *n* = 1916), general practitioner (74%. *n* = 1747), nurse (49%, *n* = 1162) and finally the psychologist, who garners only 12% of preferences (*n* = 292).

In line with previous results, dividing women between Group A and Group B regardless the age of the respondents (Table 3) and evaluating the approach toward self-examination between the two surveyed groups again shows more participation by Group B (60%) than Group A (25%), (*p* < 0.01). Women in Group B feel more conscious about performing BSE properly than women in Group A (*p* < 0.05). In fact, the greatest justification for not performing BSE related precisely to the fear of not knowing how to perform it correctly (35% Group A and 14% Group B) (*p* = 0.01).

Interestingly, this trend appears to be associated with age (*p* < 0.001), educational level (*p* < 0.001), and geographic area of origin (*p* < 0.001) (Table 1). Also associated with age, education level, and sometimes geographic area were certain concepts such as considering BSE embarrassing, time-consuming, privacy-consuming, less important than other problems, or deemed difficult because of too large breasts (Table 1).

A significant difference also relates to remembering to have checkups. While it is understandable that only a relatively small %age of women who have not had cancer remember to do checkups (26%), it is serious that only 45% of women in whom cancer was diagnosed remember to always do them, despite the risk of recurrence.

Another statistically significant difference (*p* < 0.001) concerns the need for clarification regarding breast self- examination. Our data show higher participation in screening and self-examination by women with previous breast cancer, while the %age of women in the general population needing further information about it is higher (87% vs 64%). Among the reference figures considered useful for the purpose of self-palpation, there is not much statistical difference between the two groups, with the exception of the general practitioner, who is considered more useful by women in Group A than those in Group B (74% vs 59%) (*p* < 0.001), probably due to the effectiveness of the information.

Finally, Sect. 4 investigates the knowledge and use of BSE-dedicated apps, which were used most in the pandemic period by COVID-19. Only 2% (*n* = 50) admit using them. However, the %age of those who say they use some apps including BreastTest, Igyno or Breast Cancer Indicators are still less than 3%. There is also no statistical difference between the two groups of women analyzed.

## Discussions

The aim of the study was to investigate Italian women's attitudes toward clinical breast cancer screening. Women's knowledge, use, and emotional approach toward breast self-examination as an investigative tool and the possible use of apps dedicated to it were also investigated.

Section 2 of the study investigated adherence to free screening programs aimed at women in the 50–69 age group and offered by the NHS. Incredibly, despite the fact that almost all over-50 women said they were aware of the existence of free mammography screenings, non-adherence is high. Participation in free screening also correlates with educational level and geographic area. Southern Italy and the islands were in fact characterized by lower participation in screening programs. The data appears to be in line with the national 2020 data, where in the South and Islands the crude rate of invitation adherence appears to be low (29%), compared to the North (58%) and the Center (44%).

According to the National Screening Observatory, from 2014 to the present, there has been a gradual decrease in the raw adherence to the invitation to free screening, also due to the pandemic in which the adherence dropped from 86 to 64% [12]. Also in this study, 30% of respondents say that screening programs were interrupted by the COVID-19 pandemic. It is certain that the pandemic affected many women's choice not to undergo breast screening and even surgery, partly because of fear of infection, as shown by the results of an Italian study, conducted by Tor Vergata University [21].

It is certainly reasonable also to note that women who had episodes of breast cancer compared with others are also getting screened at least once a year in younger age groups. The age at which screening should begin, however, is much debated. The Guidelines Development Group (GDG) of the European Commission Initiatives on Breast and Colorectal Cancer (ECIBC) suggests that mammography screening should not be implemented for women younger than 45 years of age, recommending biennial or triennial screening for them depending on the age groups [7]. However, this suggestion differs from what some American organizations such as the American College of Radiology and the National Comprehensive Cancer Network advocate, instead recommending annual screening starting at age 40 [8]. There were even some women in our study who admitted to undergo examinations every six months, especially those with previous cancer (*p* = 0.05). However, annual or even less than annual frequency appears to be discouraged [7]. The risk of over-diagnosis, i.e., detecting tumor formations that are treated pharmacologically—and would instead remain indolent if left untreated—carries negative psychological consequences associated with this course of ascertainment [11].

There is also significant difference between the types of examinations conducted in the A and B groups. As obvious, women in the general population undergo clinical examinations significantly less than women with prior cancer (*p* < 0.001). Among these examinations, mammography is believed to be the most widely used screening for breast cancer detection and contributes to reducing mortality [15]. Also in our results, mammography is ranked first among the most frequently performed examinations by women with previous cancer (81%). It is interesting to highlight that there are many women in the 41–50 age group who undergo mammography despite the fact that they are not yet called by SSN. This could result—aside from the natural tendency for greater scrutiny with increasing age—also from increased awareness by the physician and/or peer social environment, given the higher prevalence of breast cancer in young women with a family history of breast cancer [10].

Ultrasound is considered the first level of imaging to be performed especially in young women. Indeed, our data also showed that 100% of women under 30 years old with previous cancer had at least one ultrasound. In contrast, MRI is not recommended as a routine examination because of the higher rate of false-positive results and high cost. This is in line with the only 2% of women in the general population who reported undergoing MRI. In the case of women with cancer, however, 31% claimed to have undergone MRI. This %age could be traced to the use of MRI in selected cases not only for diagnostic screening purposes but also for assessment of the true tumor extent in the preoperative setting.

Section 3 of the questionnaire investigates women's emotional approaches to BSE. Although almost all respondents to the questionnaire consider BSE a useful tool to take care of themselves (96%), it is performed by only 4% of respondents and almost half claim they do not know how to perform it correctly. This insecurity is most prevalent among southern Italian women with a college or high school degree, under age 50, and seems to diminish with advancing age. Other reasons stated: forgetting to perform it (1%), fear of an inauspicious prognosis (5%), and performing other clinical tests for the purpose of possible cancer discovering (2%).

As concerns remembering to get checkups, while it is understandable that only a relatively small %age of women who have not had cancer remember to get checkups (26%), it is, on the other hand, serious that only 45% of women in whom cancer has been diagnosed remember to get checkups, despite the risk of recurrence. One must perhaps question the effectiveness of the invitation letters sent by the NHS, which should assiduously follow up with women who have already had the disease. However, in line with previous results, women with cancer prove to be more aware than other women (*p* < 0.05) about BSE, while Group A women say they need more information about it (86%). Useful reference figures include the senologist, oncologist, general practitioner and nurse. The psychologist garners only 12% of preferences, and this confidence does not seem to change among women with prior cancer, despite the fact that it is now clear that getting cancer is a traumatic event that also affects a person's psychological dimension.

Section 4 of our study investigated the knowledge and use of dedicated BSE apps. It was found that the %age of those using them was less than 3%, even for women with previous cancer diagnosis. Therefore, it would be recommended that health planners adopt effective educational interventions to encourage all women to perform the offered screening and practice self-examination for breast cancer [3].

The results of the study must be considered taking into account some limitations. The reference sample consisted mainly of young women under 30 years of age, and only 338 women (14%) were in the 50–69 age group so the conclusions on this group of women may be less statistically significant. This limitation is surely related to the mode of administration through the telematic medium, which is probably more used by younger women. Possible information bias may be due to a reluctant attitude to declare and therefore admit a lack of knowledge.

## Conclusions

The results obtained, confirming previous studies in the literature, show poor adherence and lack of knowledge about the modalities, timing and benefits of screening programs, especially by women in the general population compared with those who had cancer before. The need to provide women with more information to ensure better access to screening has also emerged. Also on the topic of self-examination, very few women perform it and often do not know how to do it correctly, despite the fact that almost all of them consider BSE a benefit for self-care and a practice no less important than other clinical examinations. In light of all the findings of this study, there is an urgent need to improve disclosure regarding the benefits of preventive, evidence-based practices in order to increase women's confidence in screening and consequently their increased adherence. It would also be appropriate to introduce educational programs to promote the proper performance of self-examination: since this procedure can be performed as early as 20 years of age, it would also be useful to organize educational and informational projects in schools/universities that stimulate young women to become more aware of their breast health. Finally, it would be desirable to promote the use of technology and to encourage the participation of nurses in training courses that would enable them to educate women in the practice of prevention, particularly breast self-examination, fostering a better reputation of the nursing figure in the field of prevention.

## Data Availability

Dataset can be accessed by contacting the Corresponding author.

## Appendix 1

Tables

**Table 2:** Sect. 2 of the questionnaire responses of the adult female population aged 50–69 among subjects who had not been diagnosed with breast cancer (Group A, *n* = 278) and subjects who had already been diagnosed with breast cancer (Group B, *n* = 60). Differences in response between the two Groups were assessed. A *p* value < 0.05 was considered statistically significant (**p* < 0.05; ***p* < 0.01; ****p* < 0.001)

Questionnaire items	Group A Women in the general population aged 50–69 (*n* = 278) *N* (%)	Group B Women with cancer aged 50–69 (*n* = 60) *N* (%)	*p-value*
Section 2: Participation in screening programs			
Have you ever undergone clinical screening for early detection of breast cancer?			
Never	16 (6%)	4 (7%)	< 0.05*
Rarely	15 (5%)	2 (3%)	
Occasionally	38 (14%)	7 (12%)	
Often	68 (24%)	10 (17%)	
Always	141 (51%)	37 (62%)	
If yes, please indicate the frequency			
I have never had a screening exam	13 (5%)	4 (7%)	0.54
Every two years	125 (45%)	15 (25%)	
Once a year	131 (47%)	33 (55%)	
Every six months	7 (2%)	8 (13%)	
Once a month	2 (1%)	0	
Have you ever taken advantage of the region's free checkups?			
No	77 (29%)	20 (33%)	0.40
Yes	195 (69%)	40 (67%)	
Missing	6 (2%)	0	
Have you ever had a biopsy?			
No	138 (50%)	13 (22%)	< 0.001***
Yes	27 (10%)	29 (48%)	
Missing	113 (40%)	18 (30%)	
Have you ever had a mammogram?			
No	5 (2%)	1 (2%)	0.30
Yes	265 (95%)	58 (97%)	
Missing	8 (3%)	1 (2%)	
Have you ever undergone ultrasound?			
No	45 (16%)	6 (10%)	0.09
Yes	184 (66%)	51 (85%)	
Missing	49 (18%)	3 (5%)	
Have you ever undergone MRI?			
No	148 (53%)	21 (35%)	< 0.001***
Yes	6 (2%)	19 (33%)	
Missing	124 (45%)	20 (32%)	

2 and

**Table 3:** Questionnaire responses of the adult female population among subjects who had not been diagnosed with breast cancer (Group A, *n* = 2235) and subjects who had already been diagnosed with breast cancer (Group B, *n* = 140). Differences in response between the two Groups were assessed. A *p* value < 0.05 was considered statistically significant (**p* < 0.05; ***p* < 0.01; ****p* < 0.001)

Questionnaire items	Group A Women in the general population (*n* = 2235) *N* (%)	Group B Women with cancer (*n* = 140) *N* (%)	*p-value*
Section 2: Participation in screening programs			
Q1. Have you ever undergone clinical screening for early detection of breast cancer?			
Never	1183 (53%)	14 (10%)	< 0.01**
Rarely	166 (7%)	8 (6%)	
Occasionally	272 (12%)	13 (9%)	
Often	269 (12%)	36 (26%)	
Always	345 (15%)	69 (49%)	
If yes, please indicate the frequency			
I have never had a screening exam	1204 (54%)	13 (9%)	0.05*
Every two years	390 (17%)	23 (16%)	
Once a year	585 (26%)	78 (56%)	
Every six months	52 (2%)	24 (17%)	
Once a month	4 (0%)	2 (1%)	
Q2. Have you ever taken advantage of the region's free checkups?			
No	1557 (70%)	467 (48%)	< 0.001***
Yes	419 (19%)	71 (51%)	
I don't know what this is about	259 (12%)	2 (1%)	
Q3. Have you ever undergone a biopsy?			
No	1012 (45%)	38 (27%)	< 0.001***
Yes	91 (4%)	58 (41%)	
Missing	1132 (51%)	44 (31%)	
Q4. Have you ever undergone mammography?			
No	664 (30%)	11 (8%)	< 0.001***
Yes	690 (31%)	113 (81%)	
missing	881 (39%)	16 (11%)	
Q5. Have you ever undergone ultrasonography?			
No	536 (24%)	8 (6%)	< 0.001***
Yes	858 (38%)	121 (86%)	
missing	841 (38%)	11 (8%)	
Q6. Have you ever undergone MRI?			
No	1033 (46%)	47 (34%)	< 0.001***
Yes	50 (2%)	43 (31%)	
missing	1152 (52%)	50 (36%)	
Q7			
No			
Yes			
Missing			
Section 3: Approach toward self-examination (BSE)			
Q8. How often do you perform breast self- examination?			
Never	438 (20%)	7 (5%)	< 0.01**
Rarely	545 (24%)	22 (16%)	
Occasionally	690 (31%)	27 (19%)	
Often	495 (22%)	65 (46%)	
Always	67 (3%)	19 (14%)	
Q9. If you do not perform it, state the reason			
I perform it	895 (40%)	94 (67%)	0.01**
I perform other medical examinations	30 (1%)	6 (4%)	
I don't remember to run it	416 (19%)	9 (6%)	
Fear of ominous prognosis	105 (5%)	12 (9%)	
I don't know how to execute it correctly	788 (35%)	19 (14%)	
Missing	1 (0%)	0	
Q10. When I perform self-examination I am taking care of myself			
Strongly agree	1329 (59%)	93 (66%)	0.78
agree	806 (36%)	43 (31%)	
uncertain	95 (4%)	4 (3%)	
disagree	3 (0%)	0	
strongly disagree	2 (0%)	0	
Q11. Self-palpation is embarrassing			
Strongly agree	30 (1%)	3 (2%)	< 0.05*
agree	86 (4%)	7 (5%)	
uncertain	140 (6%)	3 (2%)	
disagree	787 (35%)	55 (39%)	
strongly disagree	1192 (53%)	72 (51%)	
Q12. Self-palpation takes too long			
Strongly agree	11 (0%)	1 (1%)	0.08
agree	52 (2%)	1 (1%)	
uncertain	292 (13%)	10 (7%)	
disagree	1073 (48%)	63 (45%)	
strongly disagree	807 (36%)	65 (46%)	
Q13. It is difficult to remember to do breast checks			
Strongly agree	58 (3%)	1 (1%)	< 0.05*
agree	344 (15%)	13 (9%)	
uncertain	371 (17%)	11 (8%)	
disagree	880 (39%)	52 (37%)	
strongly disagree	582 (26%)	63 (45%)	
Q14. I have no privacy to perform the breast check			
Strongly agree	9 (0%)	1 (1%)	0.05*
agree	83 (4%)	2 (1%)	
uncertain	145 (6%)	7 (5%)	
disagree	937 (42%)	51 (36%)	
strongly disagree	1061 (47%)	79 (56%)	
Q15. My breasts are too big by self- examination			
Strongly agree	21 (1%)	2 (1%)	0.05*
agree	76 (3%)	4 (3%)	
uncertain	279 (12%)	25 (18%)	
disagree	942 (42%)	64 (46%)	
strongly disagree	917 (41%)	45 (32%)	
Q16. I have more important problems than self-palpation			
Strongly agree	8 (0%)	0	0.11
agree	31 (1%)	3 (2%)	
uncertain	108 (5%)	5 (4%)	
disagree	896 (40%)	48 (34%)	
strongly disagree	1192 (53%)	84 (60%)	
Q17. Can I perform self-examination correctly			
Strongly agree	269 (12%)	30 (21%)	< 0.05*
agree	905 (40%)	60 (43%)	
uncertain	838 (37%)	38 (27%)	
disagree	171 (8%)	10 (7%)	
strongly disagree	52 (2%)	2 (1%)	
Q18. Would like more information about self-examination			
Yes	1952 (87%)	90 (64%)	< 0.001***
No	283 (13%)	50 (36%)	
Q19. Do you find the general practitioner useful for info on self-examination			
Yes	1664 (74%)	83 (59%)	< 0.001***
No	571 (26%)	57 (41%)	
Q20. Do you find the oncologist useful for info on self-examination			
Yes	1800 (81%)	116 (83%)	0.58
No	435 (19%)	24 (17%)	
Q21. Do you find the nurse useful for info on self-examination			
Yes	1104 (49%)	58 (41%)	0.081
No	1131 (51%)	81 (59%)	
Q22. Do you find the psychologist useful for info on self-examination			
Yes	278 (12%)	14 (10%)	0.50
No	1957 (88%)	126 (90%)	
Q23. Do you find the breast specialist useful for info on self-examination			
Yes	2172 (97%)	136 (97%)	1
No	63 (3%)	4 (3%)	
SECTION 4: KNOWLEDGE AND USE OF APPS DEDICATED TO BSE			
Q24. Do you know or use dedicated applications for self-palpation?			
No	2189 (98%)	136 (97%)	0.53
Yes	46 (2%)	4 (3%)	
Q25. Use BreastTest			
I do not use any application	255 (11%)	18 (13%)	0.30
Never	734 (33%)	41 (29%)	
Rarely	26 (1%)	5 (4%)	
Occasionally	13 (1%)	1 (1%)	
Often	3 (0%)	0	
Always	2 (0%)	1 (1%)	
missing	1202 (54%)	74 (53%)	
Q26. Do you use Igyno?			
I do not use any application	252 (11%)	18 (13%)	0.60
Never	725 (32%)	40 (29%)	
Rarely	31 (1%)	5 (4%)	
Occasionally	15 (1%)	0	
Often	4 (0%)	0	
Always	2 (0%)	1 (1%)	
missing	1206 (54%)	76 (54%)	
Q27. Do you use Breast Cancer Indicators?			
I do not use any application	251 (11%)	18 (13%)	0.40
Never	734 (33%)	40 (29%)	
Rarely	22 (1%)	5 (4%)	
Occasionally	13 (1%)	0	
Often	3 (0%)	0	
Always	0	1 (1%)	
missing	1212 (54%)	76 (54%)	

3
